# Peer Review of “Information Technology Ambidexterity, Digital Dynamic Capability, and Knowledge Processes as Enablers of Patient Agility: Empirical Study”

**DOI:** 10.2196/34110

**Published:** 2021-12-06

**Authors:** Joseph Walsh

**Affiliations:** 1 School of Health Information Science University of Victoria Victoria, BC Canada

**Keywords:** IT ambidexterity, dynamic capabilities, digital dynamic capability, knowledge processes, patient agility, hospitals, Information sciences, Information technology, digital health, healthcare, digital transformation, research models

*This is a peer veview of “Information Technology Ambidexterity, Digital Dynamic Capability, and Knowledge Processes as Enablers of Patient Agility: Empirical Study”*

## Round 1 Review

### General Comments

Thank you for the opportunity to review this paper [1] on the lesser known topic of information and communications technology ambidexterity. The paper is well cited, uses appropriate methods, and discusses the concepts and findings in a clear and thorough manner. The paper should appeal to a broad audience. It is a good example of the underrepresented information and communications technology–centered literature in health care.
